# Predicting strike susceptibility and collision patterns of the common buzzard at wind turbine structures in the federal state of Brandenburg, Germany

**DOI:** 10.1371/journal.pone.0227698

**Published:** 2020-01-24

**Authors:** Anushika Bose, Tobias Dürr, Reinhard A. Klenke, Klaus Henle

**Affiliations:** 1 UFZ–Helmholtz Centre for Environmental Research, Department of Conservation Biology, Permoserstraße, Leipzig, Germany; 2 Brandenburg State Agency for Environment, Brandenburg State Bird Conservation Centre, Nennhausen, Germany; Michigan State University, UNITED STATES

## Abstract

With the increase in wind turbines, bird collisions have developed as a potential hazard. In the federal state of Brandenburg, Germany, despite the on-going mitigation efforts of increasing the distances of wind turbines from the breeding areas of the more severely affected populations of red kites (*Milvus milvus*), the additional detrimental influences on the buzzard populations (*Buteo buteo*) have added to the challenges for wind power expansion. Using data on the regional distribution of the buzzards, along with their carcass detections around the wind turbines (WTs), we aimed to better understand their collision distribution patterns in relation to their habitat use patterns to predict their exposure to collision risk using boosted regression trees (BRTs). Additionally, we integrated the developed collision potential map with the regional density map of buzzards to identify areas of increased strike susceptibility in turbine installations. Our study showed that the buzzard collisions were primarily concentrated at the turbines situated at sensitive distances from the edges of watercourses (>1000 metres), as well as those along the edges of grasslands (>750 metres), in the green open areas around/areas with minimal settlements (750 metres-1750 metres), and along the edges of bushlands (>1500 metres), together explaining 58% of the variance in their collision distribution. Conclusively, our study is applicable to conservation because it demonstrates the identification of potential collision areas along with the causes of the collisions, in addition to demonstrating the benefits of incorporating a species collision dataset as a proxy for species presence into species distribution models to make informed management decisions to eventually combat biodiversity loss.

## Introduction

Brandenburg is of particular interest in the context of Germany’s ambitious aims of transforming energy production towards modes of renewable energy generation in the coming decades [1]. With the aim of reducing CO_2_ emissions by 80–95% by the year 2050 compared to the level in 1990, the interim target is a 40% reduction by 2020, coupled with a share of 35% from renewables [2, 3]. In Brandenburg, wind energy in particular has been increasingly explored as a main source of renewable energy, leading to the widespread construction of wind farms in the state. On the other hand, this growing production of wind energy has been accompanied by the emergence of new conservation issues, in particular, the collision of birds through direct impacts with the turbine structures [4–9]. The mortality due to direct collisions has been identified as a major threat, especially for the large, soaring raptors, being most prone and vulnerable to collision [10–14]. In addition, these species are also characterized by long generation times and low reproductive rates, making them highly sensitive to any increase in mortality [15]. Several studies on the demographic effects of wind turbine fatalities have revealed that mortality due to wind turbines may reach levels that can threaten local populations, e.g. the Egyptian Vulture (*Neophron percnopterus*) in southern Spain [8], the Golden Eagle (*Aquila chrysaetos*) in the USA [16], and the Red Kite (*Milvus milvus*) in Germany [17]. Apart from this, the indirect effects; by means of the loss of nesting and foraging habitats add to the conservation concerns [18].

Wind energy in Brandenburg to be specific, has already had the highest energy capacity amongst the other installed renewables in the state [19–24]. However, with the increase in the numbers of wind turbines, the mortality of birds from collisions has simultaneously developed as a potential hazard in the state as well [17, 25–29]. Moreover, it is becoming increasingly difficult to identify suitable sites for installations of additional turbines in the region as the saturation point has already been achieved [30]. Therefore, the deployment of additional wind turbines in the state requires precise predictions of the bird strike susceptibilities to reduce bird collisions.

Over recent decades, environmentalists and managers have normally argued against the installation of wind farms in areas with high densities of birds. They make the simplistic assumption that the higher the abundance of individuals of a given species is at a particular site, the higher their susceptibility for collision with wind energy structures installed at that particular site [31, 32]. This assumption has been readily challenged by many researchers, since their findings show that the pre-construction bird abundances and the observed numbers of carcasses as a measure of the post-construction bird collisions through detections are not closely related [12, 33, 34]. The German State Bird Conservancies have also additionally developed recommendations in terms of the distances of wind turbines to such important bird areas as well as to the breeding sites of different species of birds [35]. In general, turbine site selection follows these recommended minimum distance of wind turbines to the breeding areas of sensitive bird species based on species-specific telemetry studies, collision data, spatial functional analyses, long-term observations and expert assessments [35]. Researchers also recommend a range of verification distances around wind farms that take into account areas in which there could be a high probability for a bird species to occur. These spaces can be derived from flight corridors, preferred hunting grounds of juveniles and breeding adults, roosting sites, certain landforms that cause favourable thermal conditions or other significant habitats for the species [35].

For the federal state of Brandenburg, a major challenge for further expansion of wind energy production has been their negative effects on the breeding populations of red kites (*Milvus milvus*) [17]; Bellebaum et al. applied a model based on systematic searches for collision carcasses around wind turbines and estimated that in Brandenburg, at least 308 red kites are killed annually due to collisions with their structures alone. With more than 50% of the world population found here, Germany has a greater national responsibility for their conservation than for that of any other bird species [17, 36, 37]. However, in Brandenburg, in addition to the red kites, other species also have a high conservation importance, e.g., the lesser spotted eagle (*Aquila pomarina*), great bustard (*Otis tarda*) common buzzard (*Buteo buteo*), and the white-tailed sea eagle (*Haliaeetus albicilla*) [35].

While the distance-based recommendations may help to protect spatially restricted species populations, the challenges differ for species like buzzards, because unlike their counterparts, buzzards occur almost everywhere in the state, making future turbine installations in Brandenburg particularly challenging [38].

Therefore, to develop conflict reduction strategies for a wide-ranging species, we examined the collision potential and the strike susceptibility of buzzards across the state. Using the ensemble method of boosted regression trees (BRTs), which is a combinational algorithm based on statistical and machine-learning techniques, that has relatively recently been applied to the world of species distribution modelling [39, 40]. First, we used this method to develop a spatially explicit collision distribution model for the species across the state by means of long-term carcass data detected around turbines in relation to distances to different land use types. With buzzards occurring almost everywhere in the state, the purpose of this study was also to identify distances of wind farms to different land use types where there is a particularly high risk of collision. Second, these critical areas were further compared to the regional densities of buzzards to generate an actual strike susceptibility model across the study region.

We expect our strike susceptibility model to be applicable at the turbine deployment sites and our working methodology to be applicable only for a case-by-case review, taking into account the different land use types, their included features, the distances to the edges of these features and detailed information regarding the target species. Since the study predominantly focuses on buzzards and only on “direct” collisions with the wind turbine structures, it captures only one of the many ecological impacts of wind energy infrastructures. Therefore, the authors would like to clearly and understandably state that despite the usefulness of their study for regional planning processes, our collision distribution and strike susceptibility models are neither a substitute for detailed population monitoring nor for site-specific Environmental Impact Assessments (EIAs) in the course of project planning and while interpreting the results of our study and it is highly necessary to adjust our recommendations made for buzzards according to the specific situations present in different study regions and to the specific situations present in these study regions. The best approach is not to expect our models to be an ultimate endpoint but instead to follow it as a guide for consultation within limited resources and should not be used as a sole decision-making tool for the selection of suitable wind turbine sites in the federal state.

## Materials and methods

### Study area

**Fig 1:**
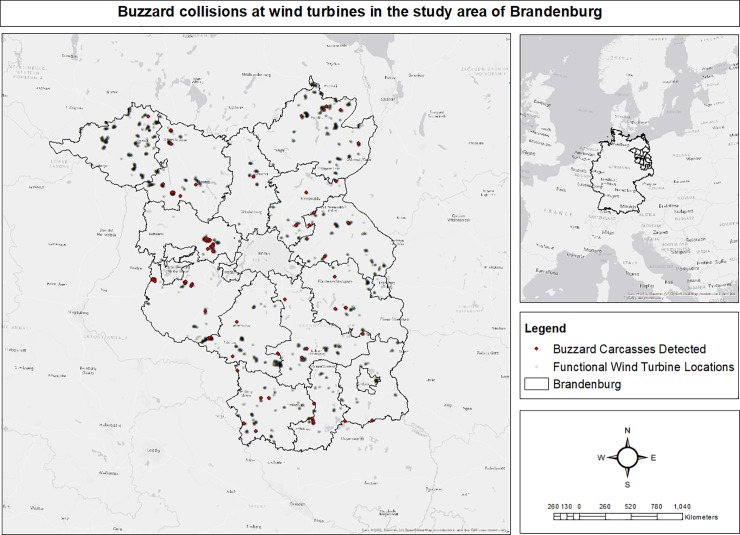
(A) Spatial locations of functional wind turbines and the wind turbines with detected Buzzard collisions in the study region of Brandenburg, Germany. (B) Number of controls per the assessed wind turbines in the study region of Brandenburg, Germany.

The federal state of Brandenburg in north eastern Germany covers an area of 29,500 km2 (Fig 1A) with a population density of only 85 people per km^2^ [1]. Brandenburg has a currently installed wind energy capacity of 5.5 GW [41] and is regarded as the world's apical region for wind energy development [42, 43]. Over recent decades, wind energy development has been rapidly paced in the state, driven by economic imperatives and aided by the sparse population density, which has led to the widespread construction of wind farms. WT structures have contributed substantially to the landscape of Brandenburg and have subsequently emerged as a new cause of bird loss [44, 45].

### Carcass search data

Spatially limited, non-uniform carcass search data of birds were available from 69 of the existing 562 windfarms (Mean: 5 functional turbines per windfarm, excluding the dismantled windfarms and wind turbines) in parts of the study region for the period 2000 to 2015 [17, 44]. From the 122 detections of exclusively buzzards, from the total number of carcasses detected, the spatial coordinate information of only these specific turbines, that reported the casualties was extracted for the purpose of our study. All carcass detections were limited by spatiotemporal inconsistencies related to researcher efficiencies due to the biases associated with the persistence times of the carcasses across the varieties of substrates [46].The largest influence although came from the differences in monitoring efforts, which ranged from only one control to many frequently and regularly controlled turbines [44] (Fig 1B). The pseudo-absence data were also biased by similar fallacies but were still numerically dominant over the presence data available across the controlled wind farms. Therefore, for the purposes of our study, we down-sized the pseudo-absence data and excluded the carcass search detections of birds belonging to the same taxonomic family as that of buzzards (i.e. *Accipitridae*) using the spatial coordinate information only from the turbines with detections of other bird groups, making neither the presence nor pseudo-absence data dominant over the other. We also ignored the estimated numbers of birds discovered in each detection and solely used the spatial coordinate information of each of the turbines where the carcasses were detected.

### Distance to edge-based land use variables (DELV)

The detailed database of land use data provided by the Biotope Type and Land Use Mapping Project of the State of Brandenburg of 2011 [47] was processed using the included features in the 12 major land use classes (Table 1) to avoid the greater degrees of inconsistencies and lack of information associated with the successive subordinate classes [44] (Fig A in S1 File). The different types of land use classes were separated; the features of each of the individual land use class were transformed to polylines and pre-processed individually to create Euclidean distances at a cell resolution of 100 metres for the whole study area with ESRI-ArcGIS version 10.1. A resolution of 100 metres was chosen to find a compromise between accuracy, the size of the raster maps, and the available computer memory or processing time. Additionally, recommendations to policymakers are rarely based on data with a resolution below 100 metres. For ease of interpretation, the created Euclidean distances were given either a negative or a positive sign to denote the distances inside and the distances outside, respectively, of the feature of a particular land use class [44]. Distance distributions of turbines under the functional wind turbine (pre-existing/with buzzard collision events/without buzzard collision events), approved and proposed wind turbine (to be installed) categories along the 12 DELVs under consideration (Fig B in S1 File).

**Table 1:** Distance to edge-based land-use variables (DELVs) used as predictors in the Federal State of Brandenburg, Germany.

Variable	Description	Coverage (%)	Variable Acronym
**Bushlands**	Deciduous bushes, field bushes, tree-lined roads, tree groups and riparian woods	**0.79**	**B**
**Fields**	Plow lands, arable lands and other farmlands	**35.11**	**F**
**Forests_forestry**	Forests and commercial forests	**35.51**	**FF**
**Flowing_watercourses**	Streaming waters, springs, small flowing rivers and channels	**0.39**	**FW**
**Green_areas_settlements**	Biotopes of green areas and open spaces including parks, gardens and village greens	**1.66**	**GS**
**Grass_forbs**	Meadows, pastures, grasslands, lawns and forb areas	**16.37**	**GF**
**Ruderal_areas**	Anthropogenic raw soil sites and ruderal areas with or without very few vegetation	**0.26**	**RA**
**Shrublands**	Dwarf shrubs, heathlands and conifer bushes	**0.35**	**S**
**Special_biotas**	Special biotopes including valleys, plantations, commercial gardens and tree nurseries	**0.87**	**SB**
**Settlements_structures**	Buildings, roads, paths, traffic and industrial areas, railroads and village like developments	**5.73**	**SS**
**Still_watercourses**	Still waters, lakes, small waterbodies, reservoirs, ponds and mine waters	**2.21**	**SW**
**Wetlands**	Mosses, swamps, sedges and peat cutting sites	**0.73**	**W**

## Methods

### Boosted regression trees

For the explanation and predictions of the collision patterns of the buzzards at wind turbines (WTs) against the distances of the turbines to the edges of various land use types, this study utilized the ensemble method of boosted regression trees (BRTs). BRTs is a machine-learning technique and builds on the concepts of decision trees and gradient boosting [39, 48, 49]. BRTs have recently gained popularity due to several advantages over traditional, frequentist statistical methods [50]. They offer high predictive accuracies and good interpretability of results, do not tend to overfit [51], are robust against missing data and collinearity of predictors, and are able to handle non-linearity and interaction effects [39, 48, 49].

Our response variable was the buzzard collisions, which were measured as the presence/pseudo-absence of buzzard carcasses around the wind turbine structures and the presence and detections of other birds through the carcass search (belonging to the taxonomic family of *Accipitridae*) around wind turbine structures as pseudo-absence data. Our predictor variables were the distances of the wind turbines to the edges of the 12 major land use classes considered (Table 1; Fig A in S1 File).

BRTs consist of two algorithms: regression trees (models that relate the response to the DELV predictors by recursive binary splitting) and boosting (adaptive method combining many of the simple models fitted iteratively in a forward stage-wise fashion to give improved predictive performance) [39]. Four parameters are important for calibrating BRTs: (*bg*) bag fraction, (*tc*) tree complexity, (*lr*) learning rate, and (*nt*) number of trees. The bag fraction specifies the share of data that is randomly withheld while fitting the model (i.e., each single tree), thereby introducing stochasticity and avoiding overfitting. The tree complexity defines the maximum order of interactions between predictors in each single tree. The learning rate reduces the contribution of each single tree to the entire model and can be interpreted as a penalizing parameter. The number of trees determines the number of single decision trees included in the model and represents the model complexity [50, 52].

We used the dismo package [53] in R [54] to implement our model and the function gbm.step, with the (*tc*) fixed at 12 (equivalent to the number of predictor variables; DELVs), the (*lr*) varied between 0.05 and 0.0005, and a default (*bf*) of 0.5 [48] to fit the models, ideally, at least 1000 trees were performed, as recommended by Elith et al. [39] was used along with the custom code [39] to generate BRT models of the collision potential for buzzards across the federal state. The model fit and predictive performances were balanced to reduce overfitting by jointly optimizing the *nt*, *lr*, and *tc* [39].

To determine the optimal number of DELVs that contributed to the response, we first ran a full model with all 12 DELVs in which a relative importance was assigned to each predictor DELV. Second, we ran another simplified model with only the highly contributing DELVs from the full model (optimal set), followed by an assessment of the response against each of them individually. We compared the goodness of fit among the models and evaluated the goodness of fit of our models using 10-fold cross-validated ROC AUC values (Receiver Operating Characteristic Area Under the Curve). [55], and the percent deviance in the cross-validation (CVdev) was also explained [52, 56] for the full and the simplified models.

We further assessed the influence of each predictor in explaining the collision patterns by calculating their relative importance in the model (number of times a variable is selected in a tree, weighted by the squared improvements, and averaged over all trees) [49]. Finally, we predicted the collision potential map for the entire study area using the model. The predictive map of the collision potential of the buzzards at the WTs *(CP)* was generated using the simplified model against the optimal set of DELVs. The predictive score of the collision potential ranged between 0 and 1 for each grid, according to the DELV characteristics of the grid cell and the model’s fitted functions. The predictive maps were validated using the test data, and their predictive capacity was determined using the AUC, sensitivity (true positive rate), and 1-specificity (false positive rate) [52].

### Regional breeding pair density and strike susceptibility

The regional density atlas of buzzards [57] was used to assess the areas of higher strike susceptibility within the assessed potential collision zones. The density map represented the number of breeding pairs (BPs) of buzzards in terms of 6 classes (i.e., BP). 1 BP, 2–3 BP, 4–7 BP, 8–20 BP, 21–50 BP, 51–150 BP) based on the paper sheet contour system of the topographical maps TK25. Most areas in Brandenburg harbour 8–20 or 21–50 BPs of buzzards on average, with lower densities commonly occurring in the fringes of the state that partly belong to its territory. The higher density areas that were categorized as having 51–150 BPs occurred mostly in the south-eastern districts of Spree-Neiße and Oberspreewald-Lausitz. For the purpose of our study, we particularly used the lower-class border of the available buzzard BP data in the state. Therefore, the lower-class border of the highest possible class of BP of buzzards available in the study area was 51 BPs. Following Torres et al [52], we calculated the strike susceptibility of buzzards at wind turbines by multiplying the assessed collision potential for the state of Brandenburg with the lower class borders of the BP of buzzards, signifying their relative density across the state using the Raster calculator function in ArcGIS version 10.1 [58].

$$\begin{array}{c} Maximum\;Breeding\;Pair\;Density\;({BPD}_{max})=51\\ Relative\;Breeding\;Pair\;Density\;({BPD}_{rel})=\\ Observed\;Breeding\;Pair\;Density\;({BPD}_{obs})/Maximum\;Breeding\;Pair\;Density\;({BPD}_{max}) \end{array}$$ (1)

$$\begin{array}{c} BPD_{rel}=\frac{BPD_{obs}}{BPD_{max}}\\ Strike\;Susceptibility\;(SC)=\\ Collision\;Potential\;(CP)\times RelativeBuzzard\;Density\;({BPD}_{rel})\times 100 \end{array}$$ (2)

$$SC=CP\times {BPD}_{rel}\times 100$$ (3)

The formula provides the strike susceptibility for buzzards at wind turbine structures and considers both the buzzard density and the influence of the landscape on collision probability. Using the potential strike susceptibility, we performed a spatial assessment of the number of existing wind turbines, number of approved wind turbines and number of proposed wind turbines within the assessed areas in the state [59].

## Results

The performance measures of the BRT model showed that the full model using all 12 DELVs, with a tree complexity (*tc*) of 12 and a default bag fraction (*bf*) of 0.5, fitted 1100 trees (*nt*) at a learning rate (*lr*) of 0.005. After the initial full model development, we further simplified the model to reduce the model complexity by sequentially dropping the least important variable with a test drop of up to 2 DELVs [39]. Between the full model and the simplified model, only 10 relatively highly influential DELVs were selected for the subsequent run of the algorithm (Table 2; Figs C1 & C2 in S1 File). Therefore, the simplified model at a tree complexity (tc) of 10 with the same default bag fraction (*bf*) of 0.5 fitted the ideally required number of trees (*nt*) of 1300 at a faster learning rate (*lr*) of 0.005. The performance of the simplified model was assessed and compared with the full model using the cross-validation running a random dataset using 30% of the occurrence points to test the model. Both models performed very well at predicting the outcomes within the training data set and resulted in satisfactory cross-validation deviance (Table 3).

**Table 2:** The relative contributions (%) of the (DELV) predictor variables for BRT full and simplified models. Developed with cross-validation on data from 332 sites and a tree complexity of 12 and 10 respectively. The full model was fitted with 12 predictors and least contributing 2 were removed and the simplified model was fit with the remaining 10 predictors; Figs C1 and C2 in S1 File.

Predictor DELV (Acronym)^1^	Full Model	Simplified Model
**FW**	11.7	12.5
**S**	10.3	11.2
**SB**	10.1	10.5
**SW**	10.0	11.1
**RA**	9.6	9.8
**GAS**	9.1	9.4
**SS**	8.7	8.9
**B**	8.1	9.1
**FF**	7.4	8.5
**GF**	7.3	8.8
**W**	4.2	
**F**	3.6	

^1^Acronyms corresponding to the predictor variables are described in Table 1.

**Table 3:** Characteristics of the BRT Full and Simplified models and their predictive performance as evaluated on the test data, within a cross validation. Both models developed with cross-validation on training data, learning rate of 0.005, using variables listed in Table 2.

Model	No. of Sites	No. of DELVs	Tree Complexity (t*c*)	Learning Rate (*lr*)	Number of Trees (n*t*)	Highest DELVs % Contribution^1^	*CV dev (%)	AUC Validation (Sensitivity, 1-Specificity)
**Full**	331	12	12	0.005	1100	**FW (11.7)**	21(0.97)	0.86 (0.69, 0.02)
						**S (10.3)**		
						**SB (10.1)**		
						**SW (10.0)**		
**Simplified**	331	10	10	0.005	1300	**FW (12.5)**	23(0.92)	0.88 (0.51, 0.01)
						**S (11.2)**		
						**SW (11.1)**		
						**SB (10.5)**		

* Mean, with standard errors in brackets

^1^Acronyms corresponding to the predictor variables are described in Table 1.

Although the validation test of the simplified model indicated a relatively low true positive rate (sensitivity = 0.51) compared to that of the full model (sensitivity = 0.69), both maintained low false-positive rates (1-specificity = 0.01 and 0.02, respectively); correspondingly, the overall discrimination of the simplified model (AUC = 0.88) was relatively equivalent to that of the full model (AUC = 0.86) (Table 3).

**Fig 2:**
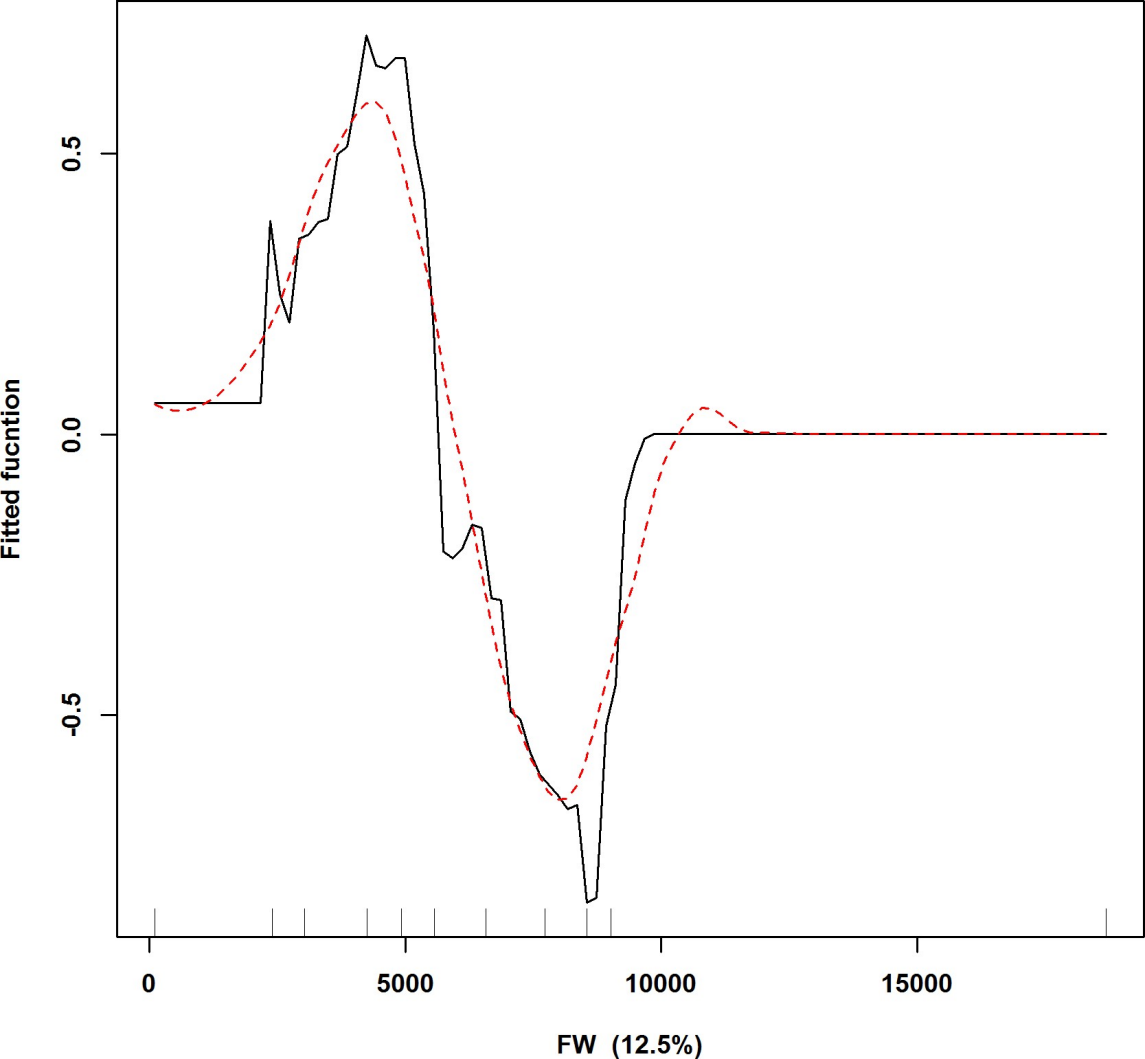
Fitted functions produced by boosted regression trees of collision potentials for buzzards at wind turbine structures depicting the marginal effect of collision possibility (y-axes) by each DELV. Contribution of each DELV is given in brackets. Rug plots show distribution of the data across distances of DELV’s in meters and are used as a measure of confidence across the shapes of the fitted functions. Signs denoting (+) are distances outside the edge of the land use variables and (-) are distances inside the edge of the land use variables.

The highly influential predictor variables according to both models were the distances to the edges of the watercourses, shrublands and special recreational parks and biotas, which together accounted for approximately 45% of the total variance in the simplified model and approximately 40% of the total variance in the full model. Among the other predictors, the distances between 1–2 km to the edges of the green and open areas around settlements (mean contribution: approximately 9.2%) contributed highly to both models. The distances to the edges of the special recreational parks and biomes up to approximately 4 km had high contributions to both the models (mean contribution: approximately 10%), followed by the distances to the edges of bushlands up to 1 km, which also showed substantial contributions (mean: approximately 8.6%). The vicinities of the open grasslands and areas with forb communities (between 0 and 500 metres) also contributed to the higher collision potential revealed by both models (mean contribution: approximately 8.05%), whereas the distances to fields contributed the least (less than 3%) (Fig 2).

**Fig 3:**
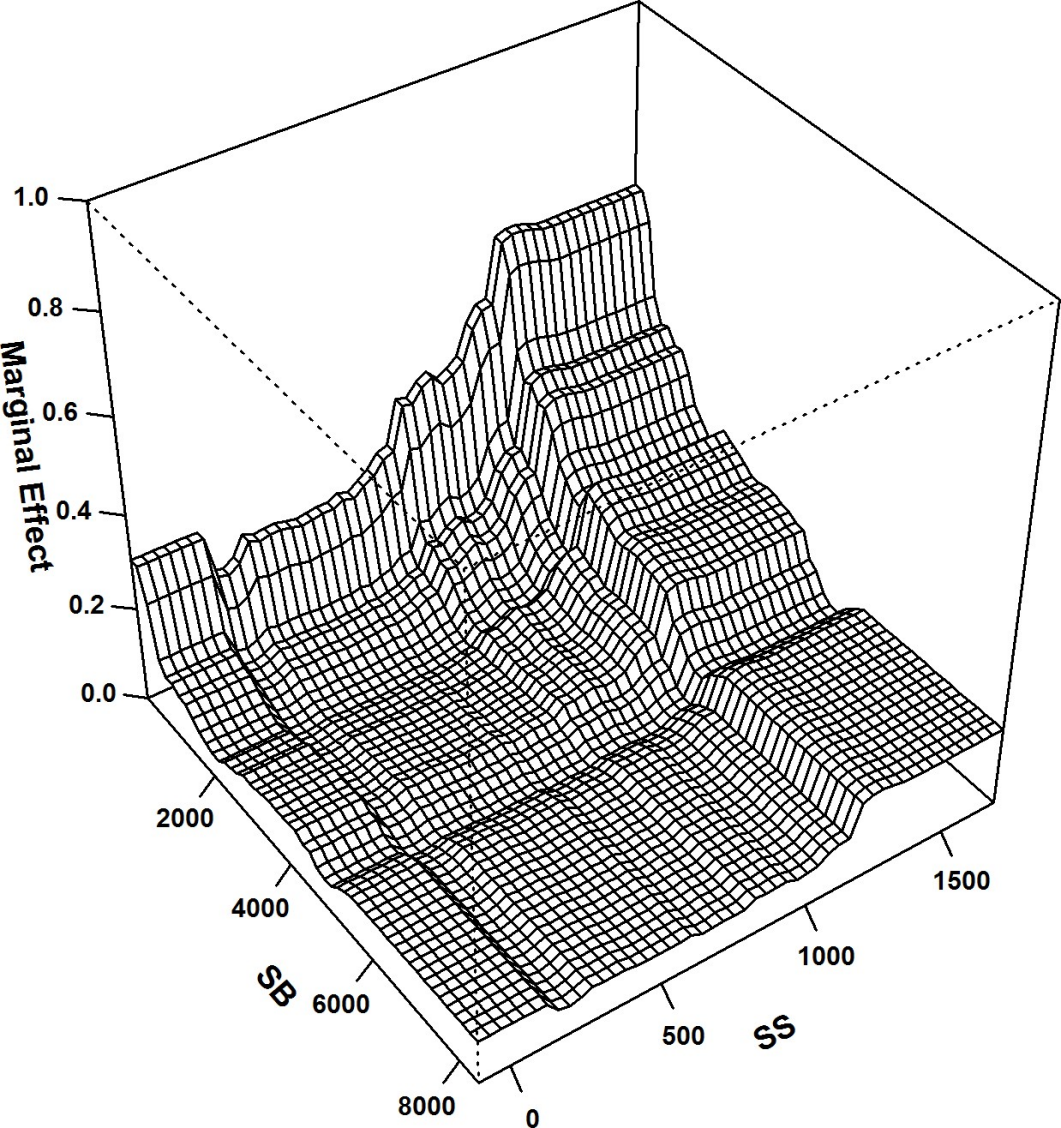
BRT 2-way: Interaction plot for distances to the edges of special biotas and the edges of settlements and structures; the most important interaction in the BRT 2-way model; interaction size = 0.58.

**Fig 4:**
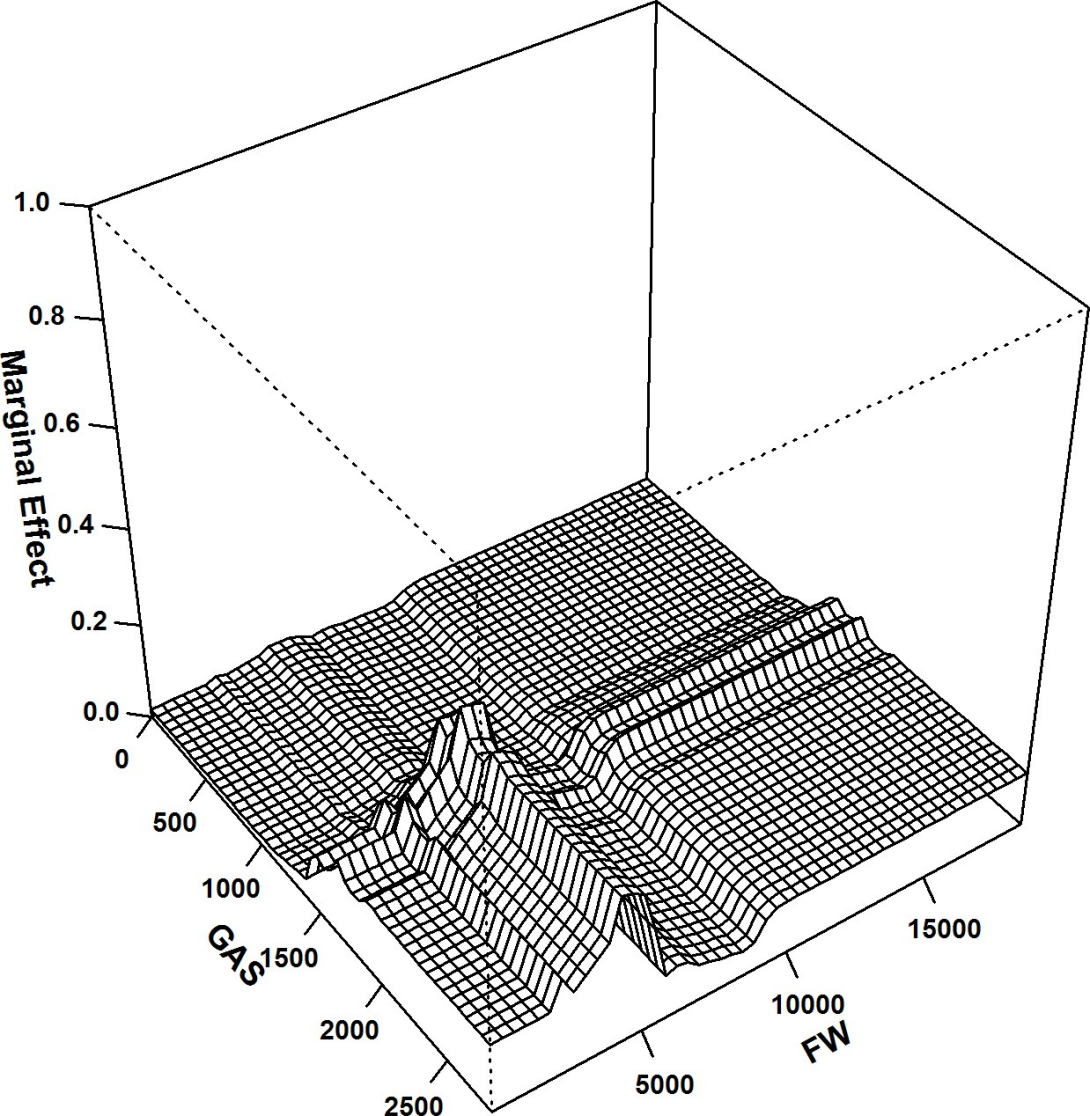
BRT 2-way: Interaction plot for distances to the edges of green areas around settlements and the edges of watercourses; the second most important interaction in the BRT 2-way model; interaction size = 0.53.

**Fig 5:**
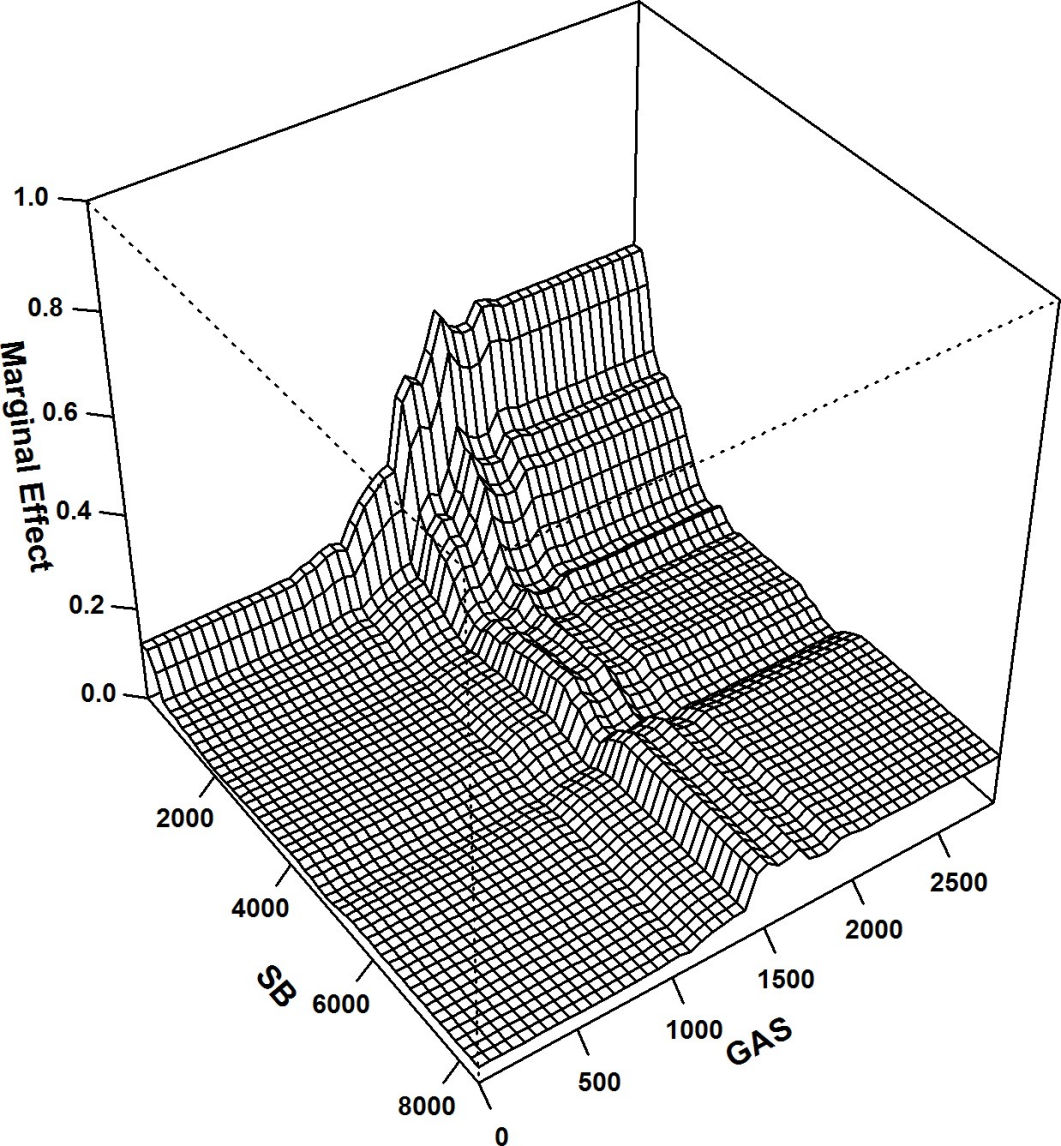
BRT 2-way: Interaction plot for distances to the edges of special biotas and the edges of green areas around settlement; the third most important interaction in the BRT 2-way model; interaction size = 0.51.

The extent of the pairwise interactions between the DELVs was also calculated; among all interactions, substantial pairwise interactions were found between the distances to the edges of the special recreational parks, biomes and settlements and the structures (variable interaction = 0.58). In addition, the distances to the edges of the green areas around settlements also showed relatively higher interactions with the distances to the edges of flowing watercourses (variable interaction = 0.53) and to the edges of the special recreational parks and biomes (variable interaction = 0.51). The variable indices of these interactions were further used for plotting their interactions to analyse the combination of the distances between the specific pairs with the highest strike risks (Fig 3, Fig 4 & Fig 5).

**Fig 6:**
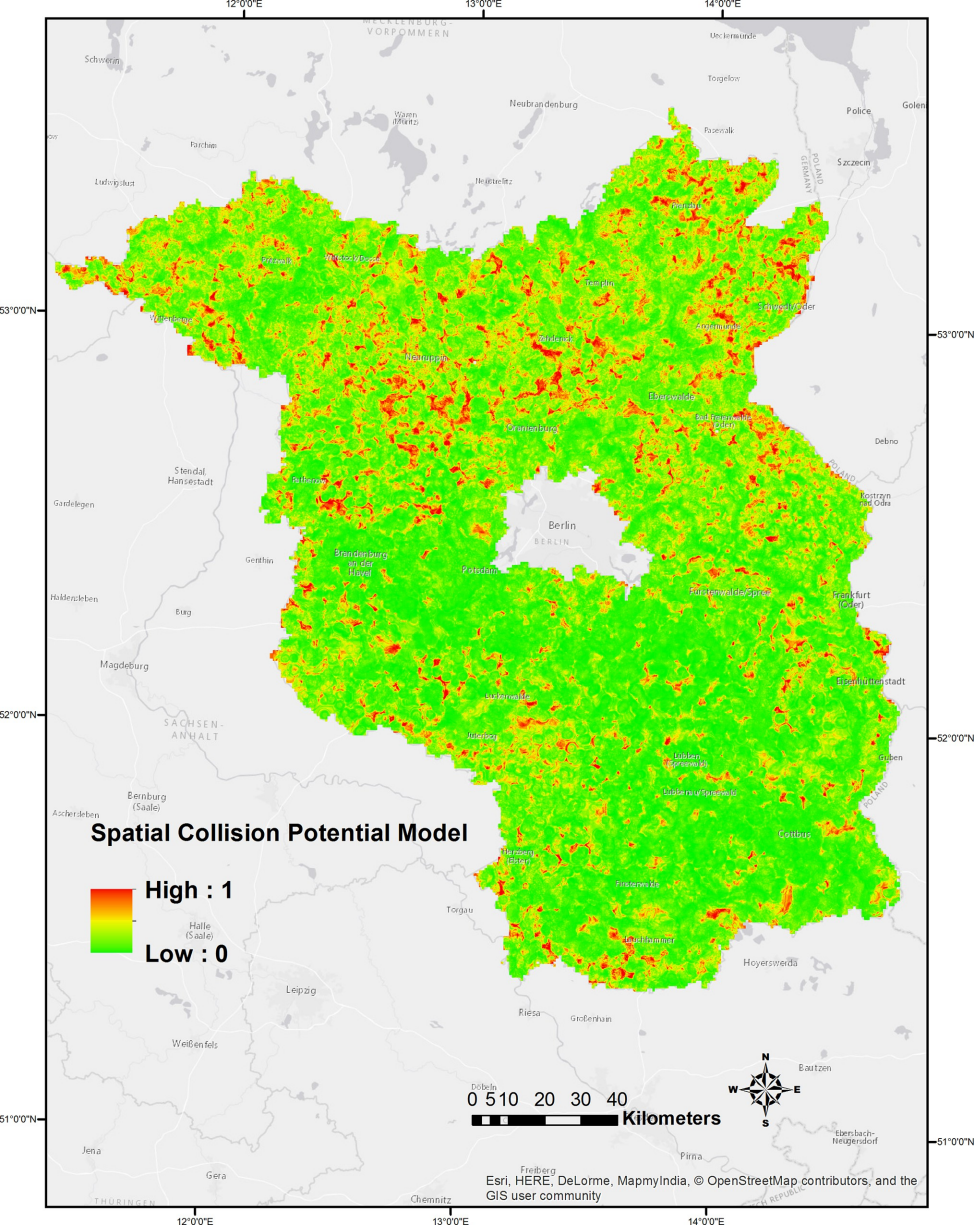
Spatial Collision Potential Model for Buzzards at WTs in the study region of Brandenburg, Germany.

**Fig 7:**
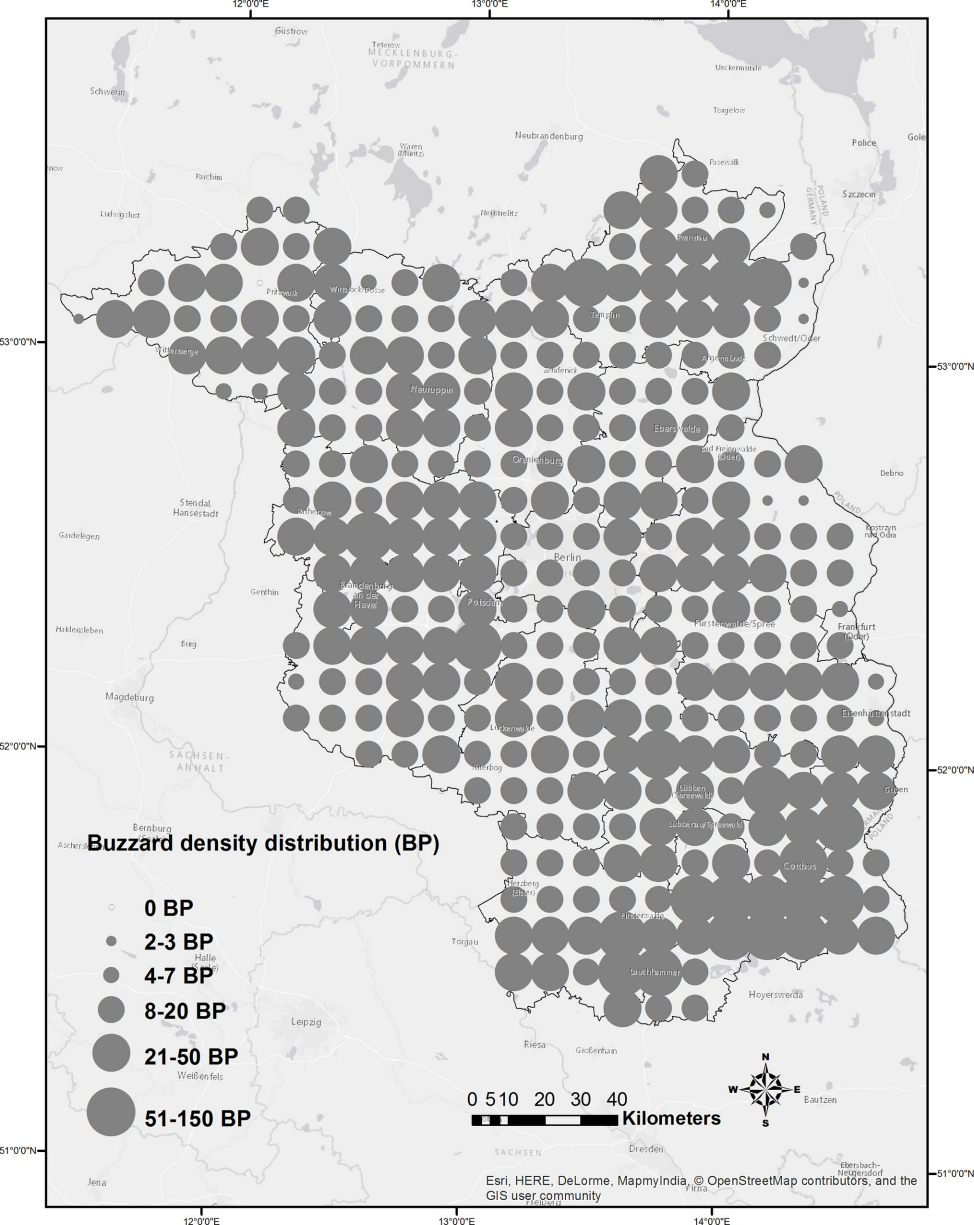
Regional densities of Buzzards in the study region of Brandenburg, Germany.

We also predicted the collision potentials for buzzards at the wind turbine structures to the DELV-based map of Brandenburg (Fig 6) using the predict function in the 'raster' package (version 2.0–12) [60] of R [54] and spatially calculated the strike susceptibility using these predicted collision potentials and the density of buzzards in the region [57] (Fig 7).

**Fig 8:**
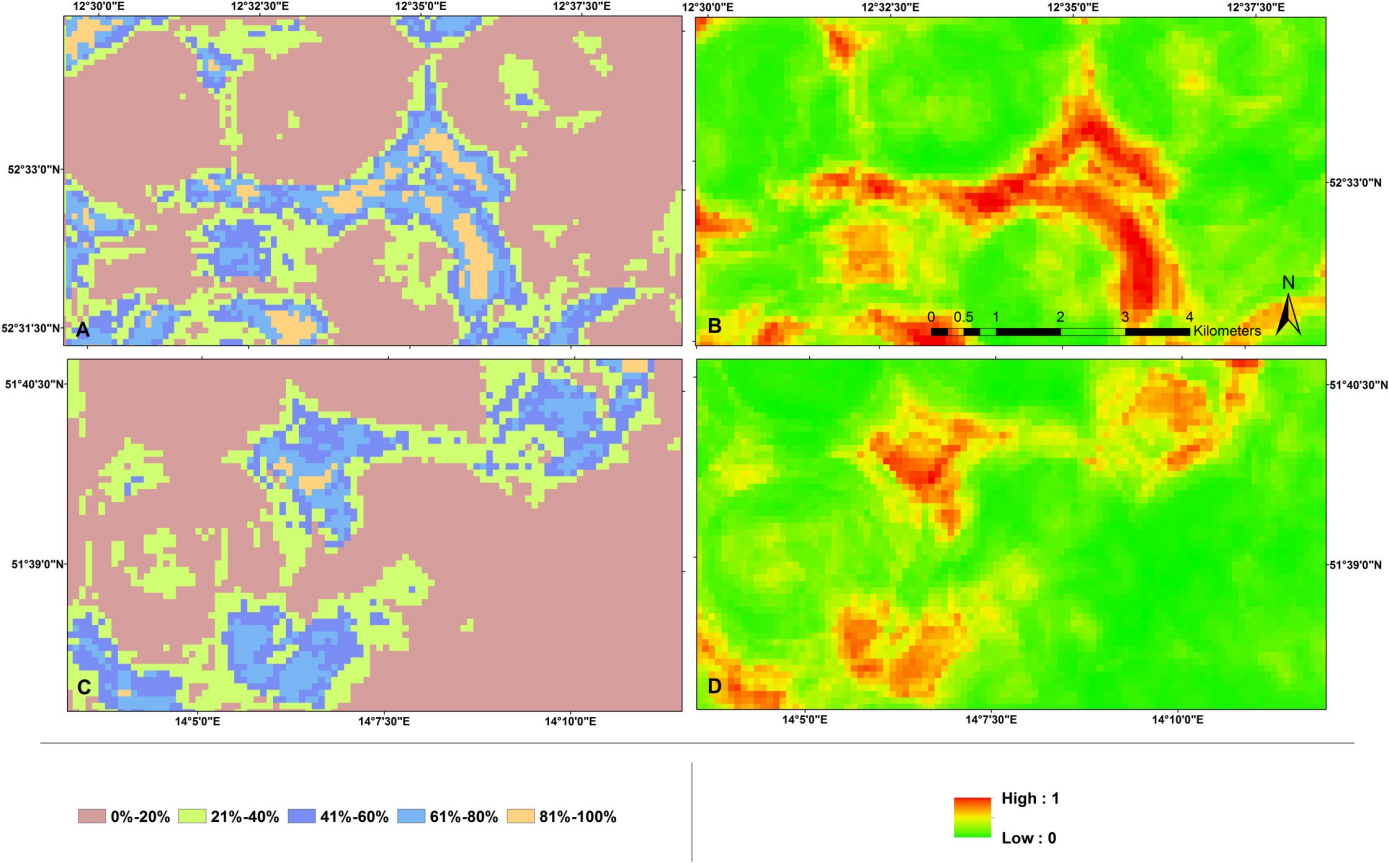
Strike susceptible locations for Buzzards at WTs in the study region of Brandenburg, Germany.

Our analyses suggest that the majority of the habitats predicted to have higher collision potentials are less susceptible to strikes (Fig 8) and that the collision potentials face relatively higher strike susceptibility (> 60%) at only some locations. In parts of the districts of Oberspreewald-Lausitz, Uckermark and Havelland, the predicted higher collision potential areas overlapped with significant densities of buzzards [57] (strike susceptibilities > 80%).

**Fig 9:**
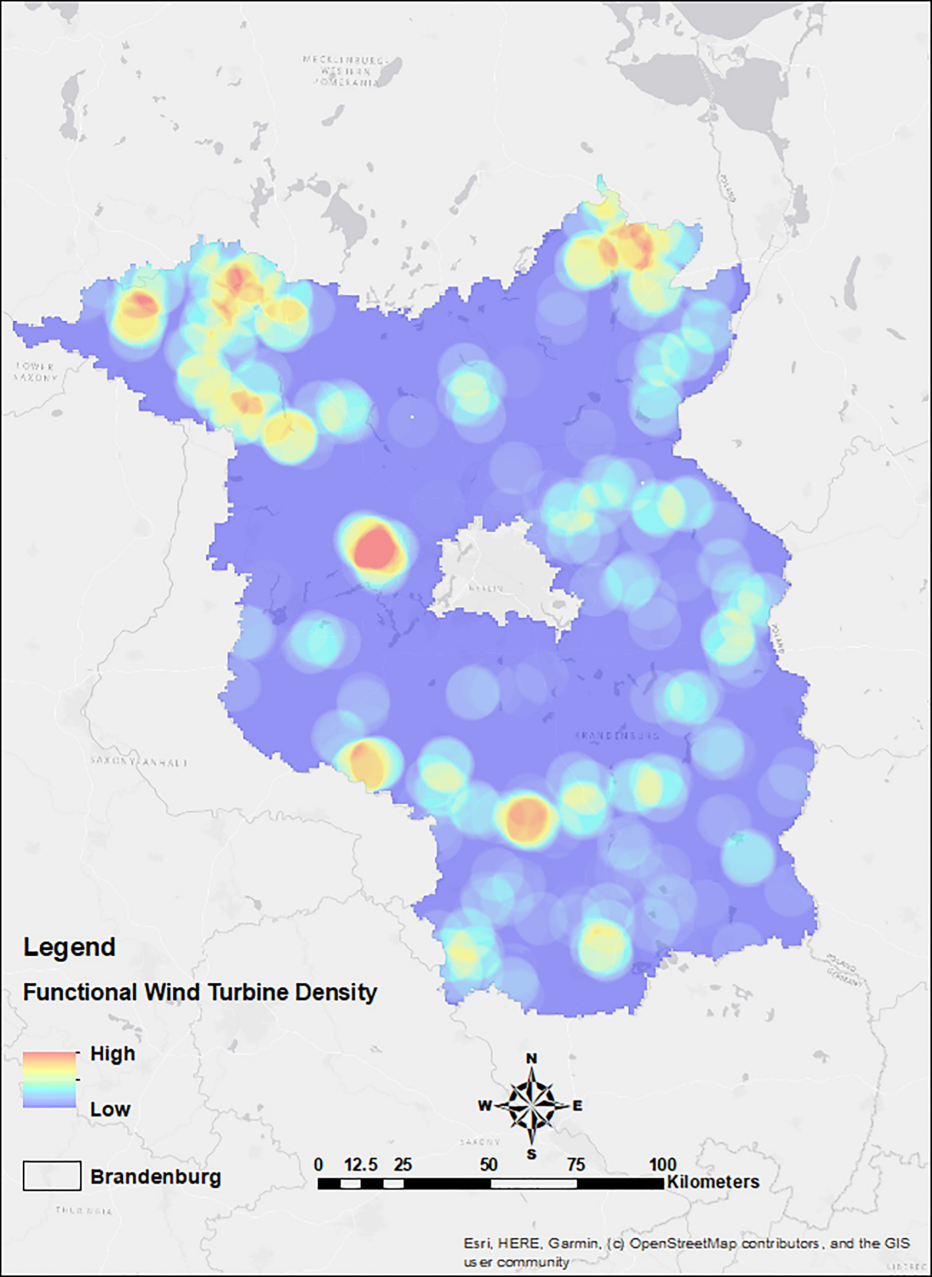
Functional wind turbine density in the study region of Brandenburg, Germany.

**Fig 10:**
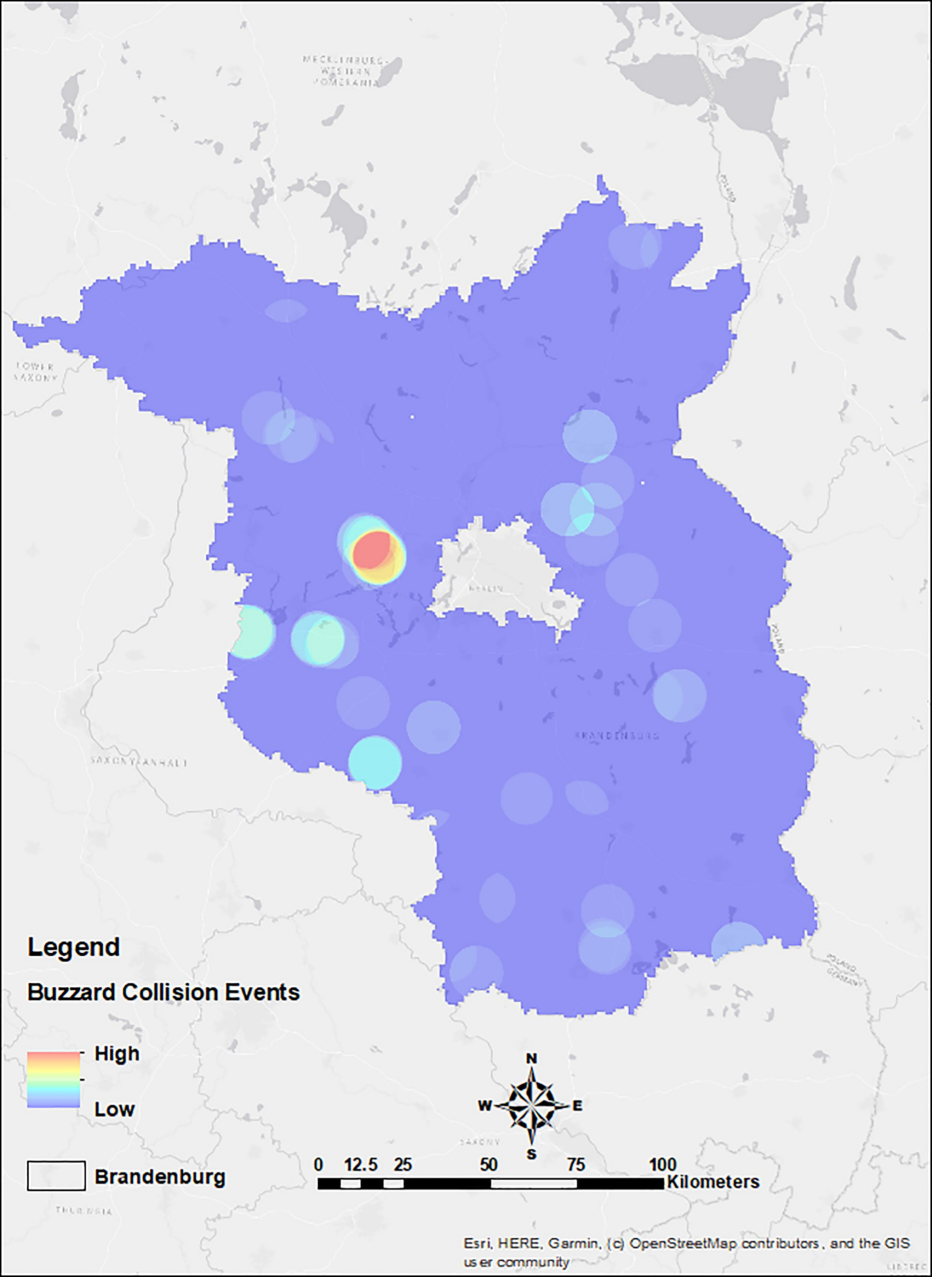
Buzzard collision events density in the study region of Brandenburg, Germany.

Moreover, we can see that buzzard pair density was higher in NE, NW, South, and West of Berlin area (Fig 7) and in Fig 1, we can see that WTs are in dense clusters in NE, NW and West of Berlin area and more equally spaced in the South. We found that functional wind turbine density coincided with the density of collision events towards the NW of the state (Figs 9 and 10).

**Fig 11:**
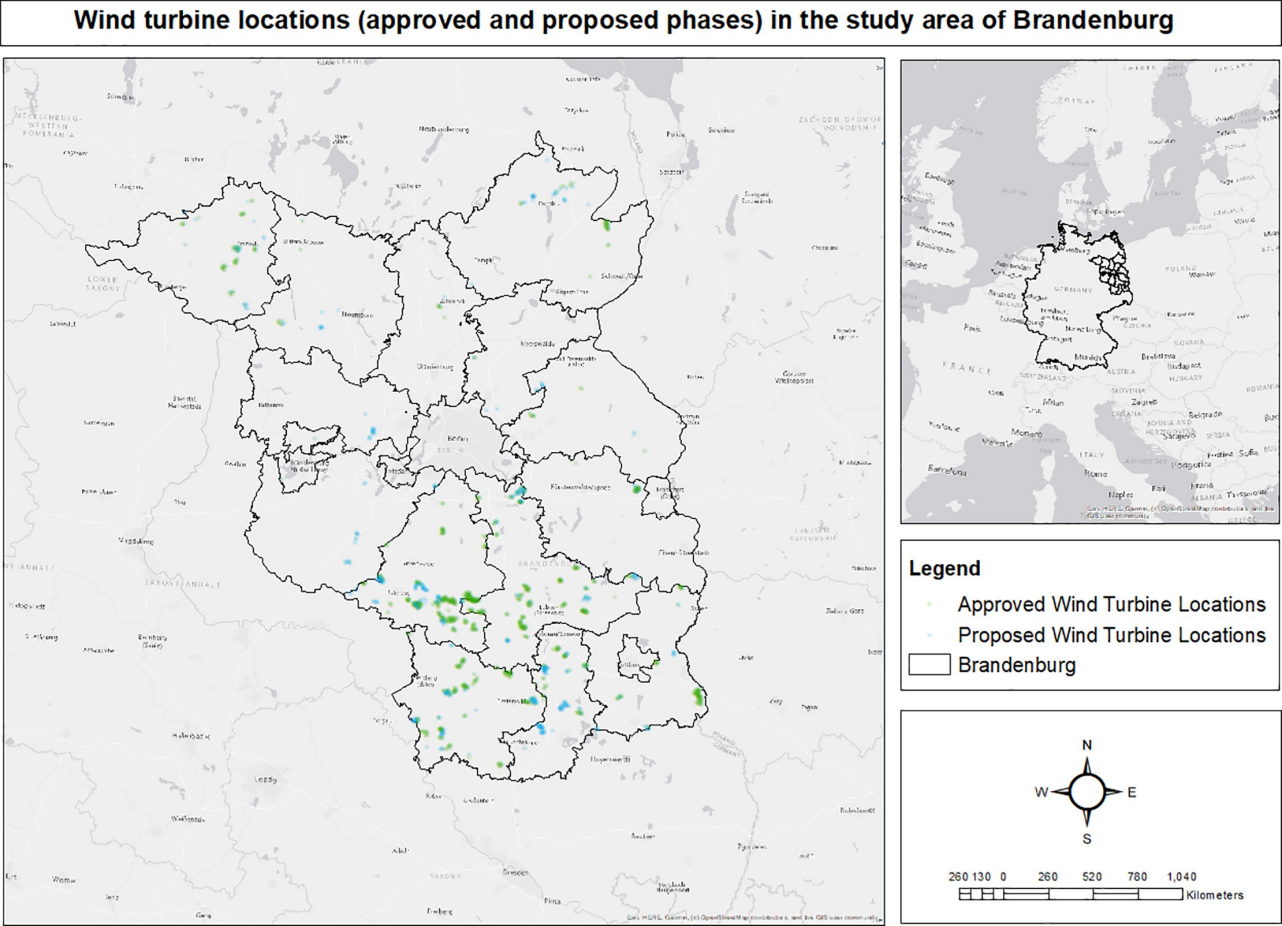
Spatial locations of the to-be deployed wind turbines in their approved and proposed phases of development in Brandenburg, Germany.

Additionally, the spatial count of the number of approved and proposed wind turbines (Fig 11) [58] to be deployed in these highly susceptible zones were also detected, and found to be merely 0.29% (4 turbines) of the total (1343 turbines) in the planned phases of wind energy development projects (Table 4).

**Table 4:** Turbines in the approved and proposed phases of development in the federal state of Brandenburg planned across the Buzzard strike susceptibility zones.

**Approved Turbines**	**Buzzard strike susceptibility at WTs**	**No. of Turbines**	**%**
	0% -20%	856	92.14
	21% - 40%	67	7.21
	41% - 60%	2	0.21
	61% - 80%	3	0.33
	81% -100%	1	0.10
**Proposed Turbines**	**Buzzard strike susceptibility at WTs**	**No. of Turbines**	**%**
	0% -20%	382	92.27
	21% - 40%	32	7.72
	41% - 60%	0	0
	61% - 80%	0	0
	81% -100%	0	0

## Discussion

Previous studies based on systematic searches of collision carcasses of birds around wind turbine structures, have not only estimated the number of birds dying as a result, but also highlighted the seasonal changes between the detections (i.e., collisions in the first place itself). Numerous studies have analysed the impacts of turbine- and the wind park overall- parameters with respect to the individual turbines (tower height, rotor radius, rotor swept area, colour, light) even the habitat parameters with respect to the positions of the turbines in the wind park (land use, distance of woodlands or water bodies to the mast foot of the turbine) and finally evaluated the accuracy of collision predictions of birds by assessing the success of future detections at the predicted locations [17, 26–29, 61–66].

On similar lines, by means of our study; we also aimed to better understand such collision distribution patterns of birds in relation to the placements of individual turbines along the various habitat use patterns around different land use types. Our endeavour was to develop conflict reduction strategies via medium of avoidance distances to direct collisions for wide-ranging species. We used this powerful tool of boosted regression trees (BRTs), which is a combinational algorithm based on statistical and machine-learning techniques, for giving general guidelines on wind power plant planning in relation to the most important landuse type variables for birds, by producing a spatially explicit map predicting their collision risk across the landscape on turbine installations. We limited the scope of our study only to that of the common buzzard (*Buteo buteo*) to predict the exposure of the collision risks to buzzards at wind energy structures. Careful site selection is crucial to reduce the risk of collision, especially in species such as the buzzard which does not seem to actively avoid wind turbine. Our study predicted the spatial patterns of wind turbine collision risks to buzzards by assessing relationships between the actual spatial occurrence of collision fatalities and bird behaviours in terms of proximities or distant preferences to different habitat features of multiple landuse types. By using the long-term carcass search data of buzzards detected around turbines; in relation to the distances of these turbines to the different land use types, we developed a spatially explicit collision distribution model for the species across the state. Additionally, the assessed collision risk areas were further compared to the regional densities of buzzards to generate their actual strike susceptibility on turbine installations across the region.

Before discussing our findings, we would like to emphasize again that our study does not rely on systematically collected, spatiotemporally homogenous bird collision data from the wind turbine structures, but on opportunistic data collected. Therefore, although our records cover a wide area, we do not know if the search regime was comparable across the study region and collected with uniform search efforts and comparable search protocols. These limitations biased the results in terms of the probability of the A.) carcass persistence times due to scavenger and predator activities and B.) the detection inadequacies of the researcher, with varying efficiencies across different substrates and species of birds involved [45]. Resulting in lack of true pseudo-absence data, which in turn led to weak and partial inferences from the predictor variables. These limitations were not unique to our study; species modelling procedures involving home range estimations, distribution evaluations and movement assessments face similar challenges regarding data issues [67–70]. These limitations should be kept in mind when interpreting the results of our study. Yet, we rule out that the carcass search operations data was biased towards wind turbines, because the all the dead bird carcasses were reported to the regional authorities and not just wind turbine collision fatalities.

Analysing the spatial information alone, we found that the density of collision events was higher in areas with higher densities of wind turbines, this effect was predominant in areas with higher regional population densities particularly (Figs 7, 9 and 10). This implies that collisions are correlated with WT density, synonymous with studies showing wind turbine densities as strong predictors of collisions, affirming the synergistic effects of wind turbine density by amplifying collision events [71], especially in areas with high buzzard density. Therefore, WT density is a critical predictor of collision, and it would have great implications on the collision risks in dense population areas. Moreover, combining collision events densities and regional species densities allowed for better predictions of collision risks (Fig 8). These results indicate that considering a combination of data on wind turbine densities along with collision events and regional population densities allows for improved assessments of collision distribution and strike susceptibilities at large spatial scales for wide-ranging birds, such as such large raptors. Therefore, our results support and encourage the use of models that use combinational data as a tool for the analysis of collision potential on larger spatial scales, as has been already done for many other bird species [7, 72–74].

However, the authors would like to clearly and understandably state that despite the usefulness of their study for regional planning processes, our collision distribution and strike susceptibility models are neither a substitute for detailed population monitoring nor for site-specific Environmental Impact Assessments (EIAs) in the course of project planning. While interpreting the results of our study it is highly necessary to adjust our recommendations made for buzzards according to the specific situations present in different study regions.

The recent shift in focus regarding the deleterious effects of wind turbines from red kites to buzzards, despite the equivalent number of collisions at WTs over the years, buzzards were not considered in the planning criteria earlier [26] because their widespread population makes them seven times as common as red kites in Germany [75]. Prohibitions to planning wind turbines with regard to species protection mostly consider species facing detrimental influences at their local population levels and exclude species that are common and widespread; collision-based losses are not considered a serious conservation issue for these species, e.g., the common buzzard [76]. However, currently in the state of Brandenburg, the inclusion of the buzzard-only criteria in the spatial planning of the turbine locations is also becoming increasingly important due to the consequential forecasted decline in the population of the species [26, 38]. Moreover, as the species is also known to occur almost everywhere in the state, selecting the lowest risk options for turbine deployment is the only strategically sound method for the continued expansion of wind power in the state.

Buzzards in general, have not shown any appreciable changes across their distribution range in Brandenburg compared to their estimated range since the mid-1990s [77], they have also not indicated avoidance behaviours with regard to wind energy structures [78]. They often approach the wind park within a few metres and use the transformers or the railings of the stairs as raised hides, making courtship flights and rare hunting flights at the hub height or above the rotors [79]. Apart from the target species, the future of wind power expansion in the state may also be unlikely, i.e. any striking changes to its spatial plan due to the innumerable number of deciding factors influencing the locations of wind turbines. As expected, the deployment of new wind turbines in most cases would either be near the vicinity of the existing turbines, adding to the output of a pre-existing wind farm, or be replaced with repowering the old wind turbines [58]. In addition to this, as our study region is in the North Eastern Germany, that has not experienced any strong land conversions over the recent decades [80], we can assume that our analyses based on the landuse type variable against the placement of turbines should not bias our results.

Buzzards are also area-sensitive species that occupy almost all habitats in the cultural landscape as long as there are suitable tree populations or artificial heights that function as breeding and nesting locations, as they prefer the use of several kinds of synonymous high natural and artificial perches [81–85], commonly at the edges of forests [84, 86, 87]. This preference has been attributed to the ease of access to the nests and to a need of unobstructed view of the surrounding landscape [84]. Therefore, maintaining a minimum distance to the fringes of the forested areas, woods surrounded by fields, tree groups and individual trees in bushlands and special recreational parks and biomes is an important planning consideration for the location of wind turbines to avoid possible collisions. Carcasses have been detected near wind turbines situated up to 750 m and 2000 m from the edges of these land use types (Fig 2).

In addition, preventative measures, to the degree that they are possible through design and effective area usage, are also recommend for the deployment of wind turbines in areas with prey attraction in the direct vicinity of the planned locations. This could include avoiding fallow lands, green and open grasslands or shrublands near the locations, as the amount of grassland and the amount of dry land are parameters strongly related to vole-hole density [87]. Although a direct connection to agricultural use does seem to exist as hunting buzzards frequently prefer fields without vegetation; it can be assumed that higher vegetation limits food visibility in an area, and thus lower and less vegetation is more favourable for food acquisition [83, 88]. Suggesting, it is essential to avoid the unintentional creation of attractive food habitats at the mast foot of the turbines due to the construction of small paved paths to access the turbines. The creation of such open areas, which have a higher edge density of greater accessibility to the potential prey base (e.g., small mammals), is widely known to increase the collision risk for the species. Additional affinities to open areas are also attributed to the promotion of courtship behaviour [83, 89].

Apart from agricultural fields, buzzard collisions also showed no affinities towards distances from watercourses. Buzzards do not select nest sites near open waters; neither the distance to the path of the watercourses influence the buzzard nest-site selection [84]. The carcasses detected at the wind turbines were situated farther than 2500 m from the borders of the flowing watercourses but closer in comparison from the borders of still watercourses; between 300 m and 1750 m (Fig 2).

The carcasses detected at the wind turbines primarily situated at distances closer to the edges of green and open areas around settlements (Fig 2), recommend that wind turbine planning should include a free approach and departure-based technique in such areas. With distances particularly between 750 m and 1750 m from the borders of green areas around settlements to be specifically avoided, i.e. avoidance of raised areas adjoining areas with open landscapes serving as possible hunting grounds, which would ultimately reduce collision risks in these areas, especially during breeding, because buzzards prefer the vicinity of their feeding areas to be in close proximity to their nesting hides [90–92].

The solutions in all cases, primarily require additional efforts in collection of the resource data. We recommend, a standardized monitoring protocol to be developed and applied prior to installations to each wind turbine construction site on a monthly basis and for a time of at least three years. Furthermore, the data from the Environmental Impact Assessment studies should be made freely accessible for monitoring on regional (state) and nationwide monitoring and research. However, post installations, there is usually relatively high-quality data for birds of prey [29, 44] despite the afore mentioned limitations due to the greater persistence times and the efficiencies of detection of their carcasses [45]. Therefore, for successful predictions and adaption of planning directives in this field, Population Viability Analyses are highly recommended [29]. In our study, the involved spatiotemporal variation was already high, which pertained to the limitations of the subsequently higher costs of data collection associated with labour requirements, further adding to the limitations. Analyses like the one we did can support the spatial planning process on regional and federal scale if not also on national scale by identification of areas with a lower risk for collision with the mentioned species. However, more research and assessment must be done with different species as well e.g. application of joint SDMs etc. These findings are particularly relevant for planners and policy makers. The differential response of birds reported suggests that it is possible to locate wind farms and to plan changes in land use in accordance with conservation interests. Depending on regional conservation priorities, it may be possible to locate suitable wind turbine sites that might affect species of lower conservation concern or even benefit those in need of conservation action. Furthermore, consideration must be given to the ecological role of these species from a wider ecological perspective.

Although we expect our approach to be applicable at the turbine deployment sites of the given study region this methodology is applicable only for a case-by-case review, taking into account the different land use types, their included features, the nearest distances to these features and the detailed information regarding the target species. Since the study predominantly focuses on buzzards and only on “direct” collisions with the wind turbine structures, it captures only one of the many ecological impacts of wind energy infrastructures. Therefore, the authors would like to clearly and understandably state that this study cannot be a substitute for an ecological impact studies at wind energy development projects. It is necessary to adjust our recommendations made for buzzards according to the specific situations present in different study regions for different species in question. Nevertheless, the best approach is not to expect the models to be an ultimate endpoint but instead to follow it as a guide for consultation within limited resources and should not be used as a sole decision-making tool for the selection of suitable wind turbine sites.

## Supporting information

S1 File Supporting figures.
**Fig A in S1 File**: **Distance to edge presentations of land-use variables in the Federal State of Brandenburg (Bose et al. 2018)** *R Development Core Team*. *2013*. *R*: *A language and environment for statistical computing*. *R Foundation for Statistical Computing*, *Vienna*, *Austria*. *ISBN 3-900051-07-0*. http://www.r-project.org/
^1^Acronyms corresponding to the predictor variables are described in Table 1.
**Fig B in S1 File: Distance distributions of turbines under the functional wind turbine (pre-existing/with buzzard collision events/without buzzard collision events), approved and proposed wind turbine (to be installed) categories along the 12 DELVs under consideration.**
**Fig C in S1 File: (1) The relative contributions (%) of the (DELV) predictor variables for BRT full and (2) simplified models.** *Developed with cross-validation on data from 332 sites and a tree complexity of 12 and 10 respectively*. *The full model was fitted with 12 predictors and least contributing 2 were removed and the simplified model was fit with the remaining 10 predictors*.
^1^Acronyms corresponding to the predictor variables are described in Table 1.
(RAR)

## References

[1] Moeller C, Meiss J, Mueller B, Hlusiak M, Breyer C, Kastner M, et al Transforming the electricity generation of the Berlin–Brandenburg region, Germany. Renewable Energy. 2014; 39–50. 10.1016/j.renene.2014.06.042

[2] BMU- The German government's energy concept- long-term strategy for future energy supply; 2010.

[3] Meyerhoff J, Ohl C & Hartje V. Landscape externalities from onshore wind power. Energy Policy. 2010; 38 (1): 82–92. 10.1016/j.enpol.2009.08.055

[4] Tikkanen H, Chiebao FB, Laaksonen T, Pakanen VM & Rytkoenen S. Habitat use of flying subadult White-tailed Eagles (*Haliaeetus albicilla*): implications for land use and wind power plant planning. Ornis Fennica. 2018; 95: 137–150.

[5] Martín B, Perez-Bacalu B, Onrubia C, de Lucas A & Ferrer M. Impact of wind farms on soaring bird populations at a migratory bottleneck. European Journal of Wildlife Research. 2018; 64: 1–10. 10.1007/s10344-018-1192-z

[6] Watson R, Kolar P, Ferrer M, Nygard T, Johnston N, Hunt G et al Raptor interactions with wind energy: case studies from around the world. Journal of Raptor Research. 2018; 52(1): 1–18. 10.3356/JRR-16-100.1

[7] de Lucas M, Ferrer M, Bechard MJ & Muñoz AR. Griffon Vulture mortality at wind farms in southern Spain: distribution of fatalities and active mitigation measures. Biological Conservation. 2012; 147:184–189. 10.1016/j.biocon.2011.12.029

[8] Carrete M, Sanchez-Zapata JA, Benitez JR, Lobon M & Donazar JA. Large scale risk-assessment of windfarms on population viability of a globally endangered long-lived raptor. Biological Conservation. 2009; 142: 2954–2961. 10.1016/j.biocon.2009.07.027

[9] Drewitt AL & Langston RHW. Assessing the impacts of wind farms on birds. Ibis. 2006; 148: 29–42. 10.1111/j.1474-919X.2006.00516.x

[10] Beston JA, Diffendorfer JE, Loss SR & Johnson DH. Prioritizing Avian Species for Their Risk of Population-Level Consequences from Wind Energy Development. PLOS ONE. 2016; 11(3): e0150813 10.1371/journal.pone.0150813 26963254 PMC4786337

[11] de Lucas M, Janss G & Ferrer M. The effects of a wind farm on birds in a migration point: the Strait of Gibraltar. Biodiversity and Conservation. 2004; 13: 395–407. 10.1023/B:BIOC.0000006507.22024.93

[12] de Lucas M, Janss G, Whitfield DP & Ferrer M. Collision fatality of raptors in wind farms does not depend on raptor abundance. Journal of Applied Ecology. 2008; 45:1695–1703. 10.1111/j.1365-2664.2008.01549.x

[13] Krone O & Scharnweber C. Two White-Tailed Sea Eagles (*Haliaeetus albicilla*) Collide with Wind Generators in Northern Germany. Journal of Raptor Research. 2003; 37(2): 174–176.

[14] Langston R & Pullan J. Windfarms and Birds: An Analysis of the Effects of Windfarms on Birds, and Guidance on Environmental Assessment Criteria and Site Selection Issues; 2003. Report by BirdLife International.

[15] Saether BE, Bakke O. Avian Life History Variation and Contribution of Demographic Traits to the Population Growth Rate. Ecology. 2000; 81: 642–653. 10.1890/0012-9658(2000)081[0642:ALHVAC]2.0.CO;2

[16] Hunt WG. Golden eagles in a perilous landscape—Predicting the effects of mitigation for wind turbine blade-strike mortality: Sacramento, California, Consultant Report to California Energy Commission under contract P500-02-043F, Public Interest Energy Research. 2002. pp. 72.

[17] Bellebaum J, Korner-Nievergelt F, Dürr T & Mammen U. Wind turbine fatalities approach a level of concern in a raptor population. Journal of Nature Conservation. 2013; 21:394–400. 10.1016/j.jnc.2013.06.001

[18] de Lucas M, Janss G & Ferrer M. Birds and Wind Farms Risk assessment and mitigation. Editorial Quercus, Madrid 2007; 280.

[19] Der NEP. Neue Netze für Neue Energien- Erläuterungen und Überblick der Ergebnisse; 2012

[20] LBV. Strukturatlas Land Brandenburg. Potsdam: Landesamt für Bauen und Verkehr. 2012. Available from: http://www.strukturatlas.brandenburg.de/

[21] Twele J, Mueller B, Moeller C & Hlusia M. Szenarioberechnung einer Strom-und Wärmeversorgung der Region Brandenburg-Berlin auf Basis Erneuerbarer Energien. Berlin: Reiner Lemoine Institut; 2012.

[22] The windpower- wind turbines and wind farms database of Brandenburg. 2012. Available from http://www.thewindpower.net/zones_en_2_brandenburg.php

[23] EEG-Anlagenregister Berlin. Deutsche Gesellschaft für Sonnenenergie e.V. 2011; Available from: http://www.energymap.info.

[24] LBV. Strukturatlas Brandenburg. Landesamt für Bauen und Verkehr. 2010. Available from: http://www.strukturatlas.brandenburg.de/

[25] Grünkorn T, Blew J, Krüger O, Potiek A & Reichenbach M. A Large-Scale, Multispecies Assessment of Avian Mortality Rates at Land-Based Wind Turbines in Northern Germany In Köppel J (eds) Wind Energy and Wildlife Interactions: Presentations from the CWW2015 Conference (2017). Springer International Publishing. 2017. pp. 43–64.

[26] Grünkorn T, Blew J, Coppack T, Krüger O & Nehls G. Prognosis and assessment of bird collision risks at wind turbines in northern Germany (PROGRESS). Final report commissioned by the Federal Ministry for Economic affairs and Energy in the framework of the 6. Energy research programme of the federal government; 2016. Available from: https://bioconsult-sh.de/site/assets/files/1575/1575.pdf

[27] Eichhorn M, Johst K, Seppelt R & Drechsler M. Model-based estimation of collision risks of predatory birds with wind turbines. Ecology and Society. 2012; 17(2): 1 10.5751/ES-04594-170201

[28] Dürr T. Vogelunfälle an Windradmasten. Der Falke. 2011; 58: 498–501.

[29] Grünkorn T, Diederich A, Poszig D, Diederichs B & Nehls G. Wie viele Vögel kollidieren mit Windenergieanlagen? Natur und Landschaft. 2009; 84 (7): 309–14. 10.1371/journal.pone.0162638

[30] Walker G, Devine-Wright P, Hunter S, High H & Evans B. Trust and community: Exploring the meanings, contexts and dynamics of community renewable energy. Energy Policy. 2010; 38: 2655–2663. 10.1016/j.enpol.2009.05.055

[31] Carrete M, Sanchez-Zapata JA, Benitez JR, Lobon M, Montoya F & Donazar JA. Mortality at windfarms is positively related to large-scale distribution and aggregation in Griffon Vultures. Biological Conservation. 2012; 145:102–108. 10.1016/j.enpol.2009.08.055

[32] de Lucas M, Janss G & Ferrer M. A bird and small mammal BACI and IG design studies in a wind farm in Malpica (Spain). Biodiversity and Conservation. 2005; 14: 3289–3303.

[33] Tikkanen H, Rytkoenen S, Karlin OP, Ollila T, Pakanen VM et al Modelling golden eagle habitat selection and flight activity in their home ranges for safer wind farm planning. Environmental Impact Assessment Review. 2018; 71: 120–131. 10.1016/j.eiar.2018.04.006

[34] Ferrer M, de Lucas M, Janss GFE, Casado E, Muñoz AR, Bechard MJ et al Weak relationship between risk assessment studies and recorded mortality in wind farms. Journal of Applied Ecology. 2012; 49: 38–46. 10.1111/j.1365-2664.2011.02054.x

[35] LAG VSW (Länderarbeitsgemeinschaft der Staatlichen Vogelschutzwarten in Deutschland). Abstandsempfehlungen für Windenergieanlagen zu bedeutsamen Vogellebensräumen sowie Brutplätzen ausgewählter Vogelarten in der Überarbeitung. 2015. Available from https://www.nabu.de/imperia/md/content/nabude/vogelschutz/150526-lag-vsw_-abstandsempfehlungen.pdf

[36] Dürr T. Zur Gefährdung des Rotmilans (*Milvus milvus*) durch Windenergieanlagen in Deutschland. Inf.-dienst Naturschutz Niedersachsen. 2009; 29: 185–191.

[37] Dürr T & Langgemach T. Populationsökologie Greifvogel- und Eulenarten. 2006; 5: 483–490.

[38] Weinhold N. Neuer Problemvogel für die Windkraft. Erneuerbare Energien. 2016. Available from: http://www.erneuerbareenergien.de/neuer-problemvogel-fuer-die-windkraft/150/434/92551/

[39] Elith J, Leathwick JR & Hastie T. A working guide to boosted regression trees. Journal of Animal Ecology. 2008; 77: 802–813. 10.1111/j.1365-2656.2008.01390.x 18397250

[40] De’ath G. Boosted trees for ecological modeling and prediction. Ecology. 2007; 88: 243–251. 10.1890/0012-9658(2007)88[243:btfema]2.0.co;2 17489472

[41] Ender C. Wind energy use in Germany- Status 31.12.2014. DEWI Magazine. 2015; 46: 26–37.

[42] Quitter J. Brandenburg is world's no.1 region for wind energy development. Wind Power Monthly. 2010 Available from http://www.windpowermonthly.com/article/1029660/brandenburg-worlds-no1-region-wind-energy-development

[43] Walker B. Power places 1. Brandenburg. Wind Power Monthly. 2010. Available from: http://www.windpowermonthly.com/article/1029660/brandenburg-worlds-no1-region-wind-energy-development

[44] Bose A, Dürr T, Klenke RA & Henle K. Collision sensitive niche profile of the worst affected bird-groups at wind turbine structures in the Federal State of Brandenburg, Germany. Scientific Reports. 8; 3777 10.1038/s41598-018-22178-z 29491479 PMC5830649

[45] Dürr T. Vogelverluste an Windenergieanlagen in Deutschland. Daten aus der zentralen Fundkartei der Staatlichen Vogelschutzwarte. Landesamt für Umwelt, Gesundheit und Verbraucherschutz Brandenburg. 2014. Available from: http://www.mugv.brandenburg.de/cms/detail.php/bb2.c.451792.de

[46] Erickson W, Wolfe M, Bay K, Johnson D, & Gehring JL. A comprehensive analysis of small-passerine fatalities from collision with turbines at wind energy facilities. PLOS ONE. 2014; 9(9): e107491 10.1371/journal.pone.0107491 25222738 PMC4164633

[47] BTLNK–Brandenburg Landesamt für Umwelt, Landwirtschaft und Geologie. Kartiereinheiten der Biotoptyen und Landnutzungskartierung Brandenburg; 2011. Available from: http://www.lugv.brandenburg.de/cms/media.php/lbm1.a.3310.de/btopkart.pdf

[48] Hastie T, Tibshirani R & Friedman JH. The Elements of Statistical Learning: Data Mining: Inferences and Predictions. Springer, New York; 2011.

[49] Friedman JH. Greedy function approximation: a gradient boosting machine. Annals of Statistics. 2001; 29: 1189–1232. 10.1214/aos/1013203451

[50] Heuck C, Brandl R, Albrecht J & Gottschalk TK. The potential distribution of the red kite (Milvus milvus) in Germany. Journal of Ornithology. 2013; 154: 911–921. 10.1007/s10336-013-0955-2

[51] Dormann CF, Elith J & Bacher S. et al Collinearity: a review of methods to deal with it and a simulation study evaluating their performance. Ecography. 2013; 36: 27–46. 10.1111/j.1600-0587.2012.07348.x

[52] Torres LG, Smith TD, Sutton P, MacDiarmid A, Banister & Miyashita T. From exploitation to conservation: Habitat models using whaling data predict distribution patterns and threat exposure of an endangered whale. Diversity and Distributions. 2013; 19(9): 1138–1152. 10.1111/ddi.12069

[53] Hijmans RJ, Phillips S, Leathwick J & Elith J. Package ‘dismo’; 2013. Available from http://cran.r-project.org/web/packages/dismo/index.html.

[54] R Development Core Team. R: A language and environment for statistical computing. R Foundation for Statistical Computing, Vienna, Austria ISBN 3-900051-07-0. 2017 Available from: http://www.R-project.org

[55] Fawcett T. An introduction to ROC analysis. Pattern Recognition Letters. 2006; 27: 861–874. 10.1016/j.patrec.2005.10.010

[56] Buston PM & Elith J. Determinants of reproductive success in dominant pairs of clownfish: a boosted regression tree analysis. Journal of Animal Ecology. 2011; 80: 528–538. 10.1111/j.1365-2656.2011.01803.x 21284624

[57] Ryslavy T, Haupt H & Beschow R. Die Brutvögel in Brandenburg und Berlin—Ergebnisse der ADEBAR-Kartierung 2005–2009. Otis Bd, 19 Sonderheft; 2011.

[58] ESRI Inc. ArcGIS 10.1. Environmental Systems Research Institute, Inc., CA, USA; 2012.

[59] LUGV–Landesamt für Umwelt, Gesundheit und Verbraucherschutz Brandenburg. Windkraftanlagen im Land Brandenburg. 2014. Interne Bezeichnung LUGV: WKA.

[60] Hijmans RJ & Etten JV. raster: Geographic analysis and modeling with raster data. R package version 2.0–12. 2012. Available from: http://CRAN.R-project.org/package = raster

[61] Weitekamp S, Timmermann H & Reichenbach H. Progress—predictive modelling versus empirical data—collision numbers in relation to flight activity in 55 German wind farm seasons. In Köppel J (eds) Wind Energy and Wildlife Interactions: Presentations from the CWW2015 Conference (2017). 2015. Springer International Publishing. pp. 242.

[62] Langgemach T & Dürr T. Informationen über Einflüsse der Windenergienutzung auf Vögel. Landesamt für Umwelt, Gesundheit und Verbraucherschutz, Staatliche Vogelschutzwarte 2015 Available from: https://lfu.brandenburg.de/media_fast/4055/vsw_dokwind_voegel.pdf

[63] Schreiber M. Artenschutz und Windenergieanlagen. Anmerkungen zur aktuellen Fachkonvention der Vogelschutzwarten. Naturschutz und Landschaftsplanung. 2014; 46(12): 361–369.

[64] Hötker H, Krone O & Nehls G. Greifvögel und Windkraftanlagen: Problemanalyse und Lösungsvorschläge. Schlussbericht für das Bundesministerium für Umwelt, Naturschutz und Reaktorsicherheit., Michael-Otto-Institut im NABU, Leitnitz-Institut für Zoo- und Wildtierforschung, BioConsult SH, Bergenhusen, Berlin, Husum; 2013 Available from: https://www.bmu.de/files/english/pdf/application/pdf/energiekonzept_bundesregierung_en.pdf

[65] Rasran L & Dürr T. Kollisionen von Greifvögeln an Windenergieanlagen—Analyse der Fundumstände In: Hötker H, Krone O, Nehls G (eds) Greifvögel und Windkraftanlagen: Problemanalyse und Lösungsvorschläge. Schlussbericht für das Bundesministerium für Umwelt, Naturschutz und Reaktorsicherheit, FKZ 0327684, Michael-Otto-Institut im NABU, Leibniz-Institut für Zoo- und Wildtierforschung, BioConsult SH, Bergenhusen, Berlin, Husum 2013 Available from: https://bergenhusen.nabu.de/imperia/md/nabu/images/nabu/enrichtungen/bergenhusen/projekte/bmugreif/endbericht_griefvogelprojekt.pdf

[66] Illner H. Kritik an den EU-Leitlinien „Windenergie-Entwicklung und NATURA 2000“, Herleitung vogelartspezifischer Kollisionsrisiken an Windenergieanlagen und Besprechung neuer Forschungsarbeiten. Eulen-Rundblick. 2012; 62: 83–100.

[67] Guillera-Arroita G. Modelling of species distributions, range dynamics and communities under imperfect detection: advances, challenges and opportunities. Ecography. 2017; 40: 281–295. 10.1111/ecog.02445

[68] Lahoz-Monfort JJ. Imperfect detection impacts the performance of species distribution models. Global Ecological Biogeography. 2014; 23: 504–515. 10.1111/geb.12138

[69] Hull CL & Muir S. Search areas for monitoring bird and bat carcasses at wind farms using a Monte-Carlo model. Australasian. Journal of Environmental Management. 2010; 17: 77–87. 10.1080/14486563.2010.9725253

[70] Kéry M. Predicting species distributions from checklist data using site-occupancy models. Journal of Biogeography. 2010; 37: 1851–1862. 10.1111/j.1365-2699.2010.02345.x

[71] Schaub M. Spatial distribution of wind turbines is crucial for the survival of red kite populations. Biological Conservation. 2012; 155: 111–118. 10.1016/j.biocon.2012.06.021

[72] Vasilakis DP, Whitfield DP & Kati V. A balanced solution to the cumulative threat of industrialized wind farm development on cinereous vultures (*Aegypius monachus*) in south-eastern Europe. PLOSONE. 2017; 12: e0172685 10.1371/journal.pone.0172685 28231316 PMC5322877

[73] Vasilakis DP, Whitfield DP, Schindler S, et al Reconciling endangered species conservation with wind farm development: cinereous vultures (*Aegypius monachus*) in south-eastern Europe. Biological Conservation. 2016; 196: 10–17. 10.1016/j.biocon.2016.01.014

[74] Reid T, Krüger S, Whitfield DP & Amar A. Using spatial analyses of bearded vulture movements in southern Africa to inform wind turbine placement. Journal of Applied Ecology. 2015; 52: 881–892. 10.1111/1365-2664.12468

[75] Bauer HG, Berthold P, Boye W, Knief P, Südbeck, et al Rote Liste der Brutvögel Deutschlands 3., überarbeitete Fassung. Ber. Vogelschutz. 2002; 3: 50.

[76] Stadt Rheinbach. Artenschutzrechtlicher Fachbeitrag zum Bebauungsplan der Stadt Rheinbach. Ing.- und Planungsbüro LANGE GbR. 65. 2015. Available from: http://www.rheinbach.de/imperia/md/content/cms121/bauenwohnenundstadtentwicklung/stadtentwicklung/181115_artenschutzrechtlicher_fachbeitrag.pdf

[77] ABBO: Die Vogelwelt von Brandenburg und Berlin. Rangsdorf; 2001.

[78] Bergen F. Untersuchungen zum Einfluss der Errichtung und des Betriebs von Windenergieanlagen auf Vögel im Binnenland. Dissertation. Ruhr Universität Bochum; 2001.

[79] Sinning F & Gerjets D. Untersuchungen zur Annäherung rastender Vögel an Windparks in Nordwestdeutschland. Bremer Beiträge für Naturkunde und Naturschutz Bd. 1999; 4: 53–60.

[80] Kuemmerle T, Levers C, Erb K, et al Hotspots of land use change in Europe. Environmental Research Letters. 2016; 11: 064020 10.1088/1748-9326/11/6/064020

[81] Mülner B. Winterliche Bestandsdichten, Habitatspräferenzen und Ansitzwartenwahl von Mäusebussard (*Buteo buteo*) und Turmfalke (*Falco tinnunculus*) im oberen Murtal (Steiermark). Egretta. 2000; 43: 20–36.

[82] Probst R. Greifvogelu¨berwinterung 1998 bis 2002 im Bleistätter Moos, Kärnten. Carinthia II. 2002; 114: 509–516.

[83] Penteriani V & Faivre B. Breeding density and landscape-level habitat selection of common Buzzards (*Buteo Buteo*) in a mountain area (Abruzzo Apennines, Italy). Journal of Raptor Research. 1997; 31(3): 208–212.

[84] Hubert. Nest-site habitat selected by Common Buzzard (*Buteo buteo*) in south western France. Journal of Raptor Research. 1993; 27: 102–105.

[85] Glutz von Blotzheim UN, Bauer KM & Bezzel E. Handbuch der Vögel Mitteleuropas. Bd. 9: Falconiformes. Aula-Verlag, Wiesbaden 1989.

[86] Graham IM, Redpatha SM & Thirgood SJ. The diet and breeding density of Common Buzzards (*Buteo buteo*) in relation to indices of prey abundance. Bird Study. 1995; 42: 165–173. 10.1080/00063659509477162

[87] Hohmann U. Status specific habitat use in the Common Buzzard (*Buteo buteo*). Raptor Conservation Today. Proc. IV World Conference on Birds of Prey and Owls. WWGBP/The Pica Press, Berlin, Germany; 1994: 359–366.

[88] Schindler S, Hohmann U, Probst R, Nemeschkal HL & Spitzer G. Territoriality and habitat use of common Buzzards (*Buteo buteo*) during late autumn in northern Germany. Journal of Raptor Research. 2012; 46: 149–157. 10.3356/JRR-11-22.1

[89] Cerasoli M & Penteriani V. Nest-site and aerial meeting point selection, nesting density and reproduction of Common Buzzards. Journal Raptor Research. 1996; 30: 130–135.

[90] Kenward RE, Clarke RT, Hodder KH & Walls SS. Density and linkage estimators of home range: nearest-neighbor clustering defines multinuclear cores. Ecology. 2001a; 82: 1905–1920. 10.1890/0012-9658(2001)082[1905:DALEOH]2.0.CO;2

[91] Kenward RE, Hall DG, Walls SS & Hodder KH. Factors affecting predation by Buzzards (*Buteo buteo*) on pheasants (*Phasianus colchicus*). Journal of Applied Ecology. 2001b; 38: 813–822. 10.1046/j.1365-2664.2001.00636.x

[92] Newton I. Population ecology of raptors, Second Ed. T. and A.D. Poyser. Berkhamsted, U.K; 1990.

