# Correction: Predicting strike susceptibility and collision patterns of the common buzzard at wind turbine structures in the federal state of Brandenburg, Germany

**DOI:** 10.1371/journal.pone.0238269

**Published:** 2020-08-26

**Authors:** Anushika Bose, Tobias Dürr, Reinhard A. Klenke, Klaus Henle

Figs 1 and 2 are incomplete. Please see the complete correct Figs 1 and 2 here.

**Fig 1:**
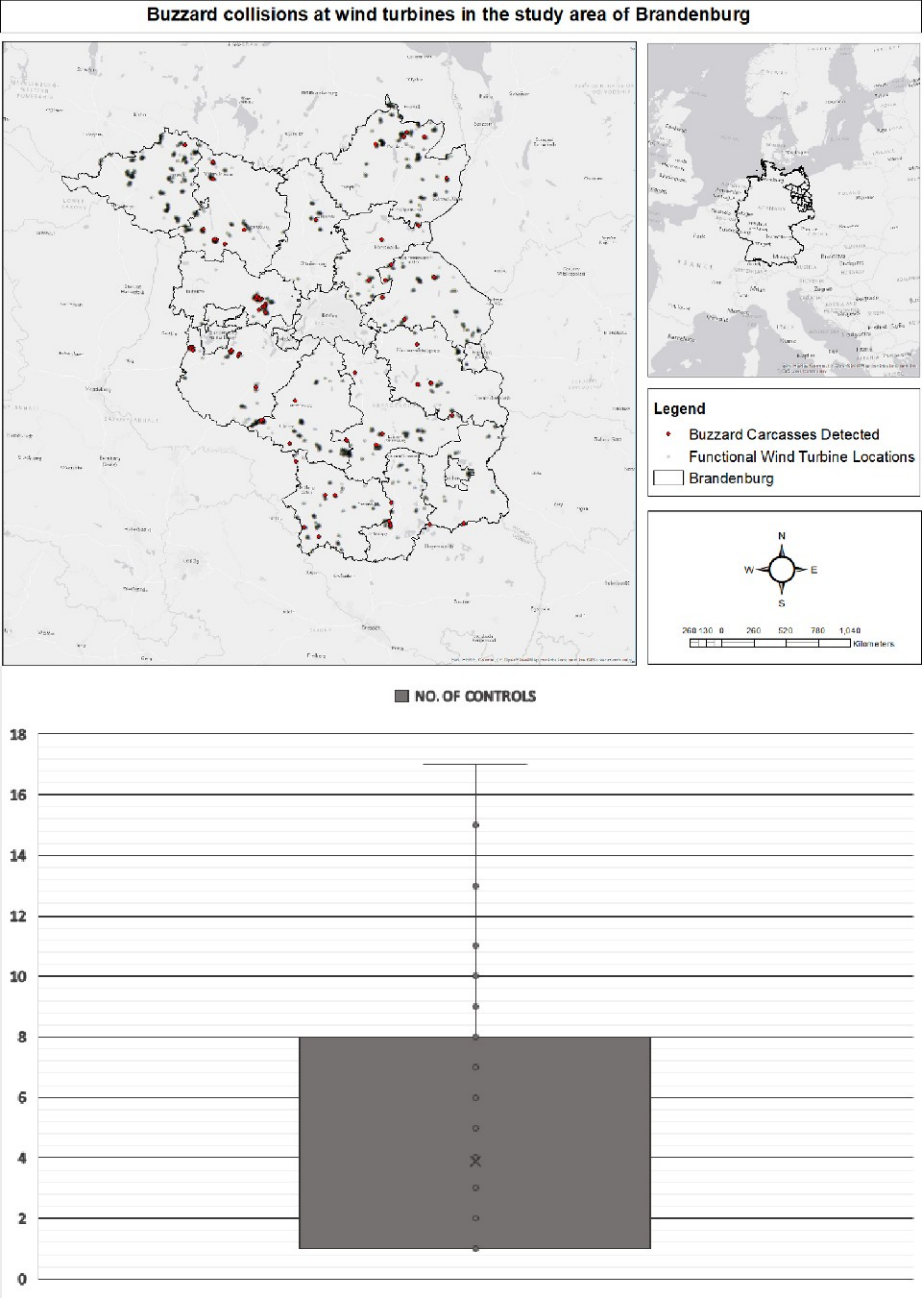
(A) Spatial locations of functional wind turbines and the wind turbines with detected Buzzard collisions in the study region of Brandenburg, Germany. (B) Number of controls per the assessed wind turbines in the study region of Brandenburg, Germany.

**Fig 2:**
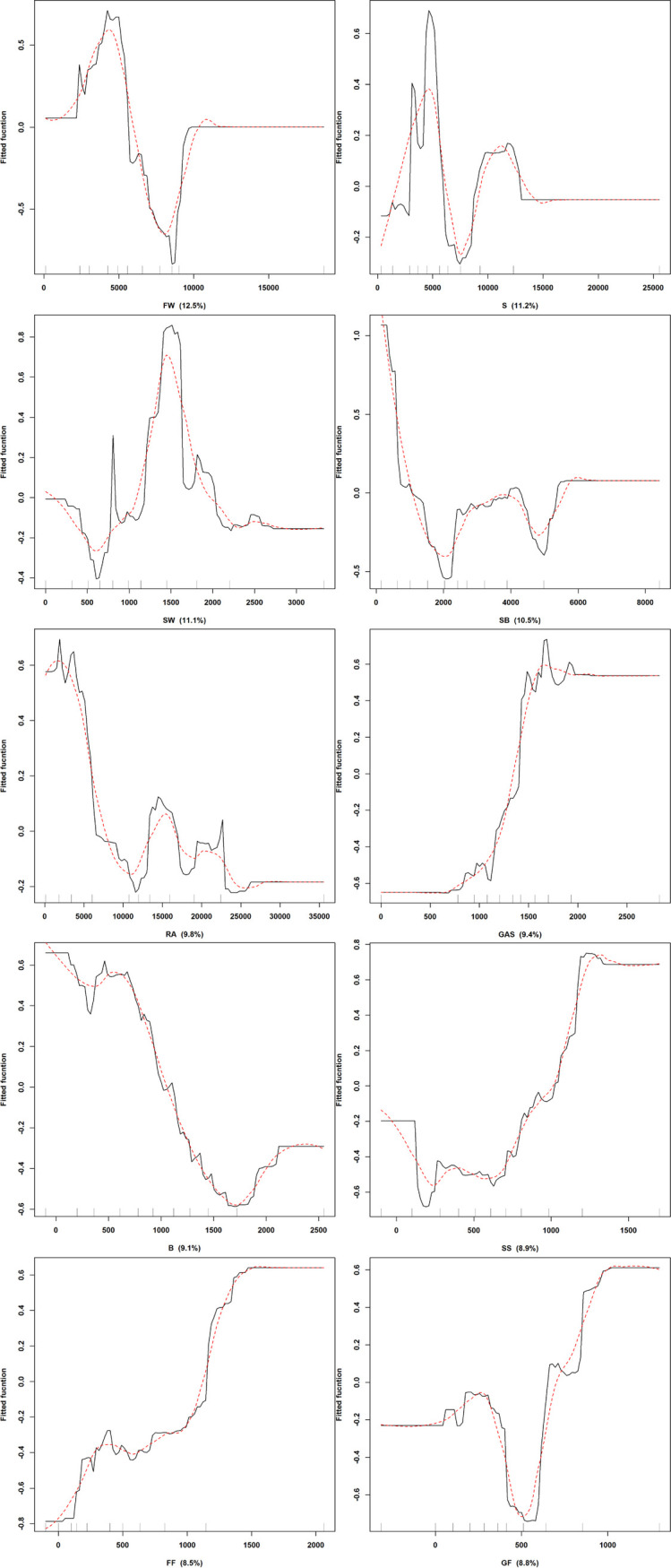
Fitted functions produced by boosted regression trees of collision potentials for buzzards at wind turbine structures depicting the marginal effect of collision possibility (y-axes) by each DELV. Contribution of each DELV is given in brackets. Rug plots show distribution of the data across distances of DELV’s in meters and are used as a measure of confidence across the shapes of the fitted functions. Signs denoting (+) are distances outside the edge of the land use variables and (-) are distances inside the edge of the land use variables.
